# Development and validation of the Griffith University Affective Learning Scale (GUALS): A tool for assessing affective learning in health professional students’ reflective journals

**DOI:** 10.15694/mep.2018.000002.1

**Published:** 2018-01-04

**Authors:** Gary D. Rogers, Amary Mey, Pit Cheng Chan, Marise Lombard, Fiona Miller

**Affiliations:** 1 School of Medicine and Health Institute for the Development of Education and Scholarship (Health IDEAS); 2 School of Pharmacy and Pharmacology; 3 School of Medicine; 4 School of Pharmacy and and Pharmacology

**Keywords:** affective learning, assessment, interpretative phenomenological analysis, professionalism, interprofessional values

## Abstract

This article was migrated. The article was marked as recommended.

Background

Assessment of health students’ attainment of cognitive and psychomotor learning outcomes is achieved through the application of well-established methods. However, for learning in the affective domain, which, in the health professions, is closely associated with the development of ‘professionalism’, assessment remains challenging as there is a dearth of validated, reliable and practical tools available. The aim of this study was to develop and test the reliability of an instrument assess for evidence of affective learning in the reflective journals of health professional students who have participated in emotionally-impactive learning experiences.

Method

Based on the findings of our earlier published work on the assessment of affective learning ( Rogers, Mey & Chan, 2017), we developed a practical tool known as the Griffith University Affective Learning Scale (GUALS). We trained a pool of learning facilitators in the assessment of affective learning and the use of the instrument. Two facilitators, in parallel, independently graded each of the daily journals of 26 medical students undertaking a week-long immersive simulation activity. Assessors were asked to rate the highest level of affective learning evident in each journal. Statistical analysis explored score distribution, means and inter-rater differences.

Results

One hundred and twenty-five journal entries (five from each of 25 students - one selected student had missing journals and was thus excluded from the analysis) were rated by a total of seven trained facilitators. Scores were normally distributed, with a mean of 4.23 (SD = 1.10) on a seven-point scale. Inter-rater absolute score concordance was seen for 45.6% of the journals, with a mean inter-rater difference of 0.56 points, maximum difference of 2.00 points and intraclass correlation coefficient of 0.86 (95% Cl: 0.80 - 0.90).

Conclusions

GUALS, when utilised by trained assessors, appears to be a reliable tool to assess for evidence of affective learning in medical students’ journals related to emotionally-impactive simulated clinical experiences. Further research should explore its utilisation in relation to other learning experiences such as real clinical setting encounters, as well as with students from other health professions and in other settings where the assessment of affective learning is important.

## Introduction

In the field of education, the idea that learning occurs within cognitive, psychomotor and affective domains was originally characterised by Benjamin Bloom and colleagues (1956) and retains currency today. The framework became known as Bloom’s Taxonomy of Learning and has been widely adapted by educators in many settings.

According to this schema, learning in the cognitive domain, which has been the traditional focus of education, concerns the acquisition of knowledge and understanding in relation to specific topics. At its higher levels, it emphasises critical thinking, analysis and the application of understanding to new problems. Psychomotor learning facilitates the development of skills, particularly physical skills. An extension of the psychomotor domain might also include the learnable practical skills required for effective communication, for example. The affective domain focuses on the development of values and attitudes through the recognition of emotional responses to learning experiences. In healthcare practice, it requires recognition of the human elements of learning and attention to practitioners’ personal emotional responses to their experiences. This enables learners to examine their own value systems and develop worldviews that are congruent with the values of their chosen profession.

Learning within each of Bloom’s domains can be characterised by hierarchical levels of achievement and in the affective domain these were first characterised in work led by Bloom’s associate, David Krathwohl ( Krathwohl, Bloom, & Masia, 1964). Krathwohl defined the levels of affective learning (from lowest to highest) as ‘receiving’, ‘responding’, ‘valuing’, ‘organising’ and ‘characterising’. At the *receiving* level, learners notice and engage with experiences that might lead to learning in the affective domain. At the *responding* level, they become aware of their own emotional responses to the learning experiences and start ‘to attach emotional significance and value to them’ ( Krathwohl et al., 1964, p. 34). At the *valuing* level learners begin to make sense of the experiences and their emotional responses in terms of their potential to impact their pre-existing values. At the *organising* level, they commence incorporation of the affective learning brought about by the experiences into their value systems and view of the world. At the highest level, *characterising*, learners have fully integrated learning from the experiences into their worldviews and resultant professional practice.

Scholarly work in health professional education has yielded a range of valid and practical techniques for the assessment of learning in the cognitive domain (such as multiple choice, short answer, extended matching and script concordance questions) and the psychomotor domain (such as the ‘Objective’ [or Observed] Structured Clinical Examination [OSCE] and the mini Clinical Examination [mini-CEX]). However, the assessment of affective learning, which we would argue is central to the acquisition of critical capabilities such as ‘professionalism’, has been much less studied and few practical methods are available to undertake it.

During 2011 and 2012, we conducted a randomised controlled trial to investigate the effectiveness of a new interactive learning model for students enrolled in our Doctor of Medicine degree. Penultimate year medical students randomised to the intervention arm were provided with the opportunity to engage in Clinical Learning through Extended Immersion in Multimethod Simulation (CLEIMS) - week-long, extended simulation activities designed to replicate their future lives as junior doctors. This included interaction with simulated patients and interprofessional learning with students from other health disciplines, interspersed with more traditional seminars and workshops on topics raised by the simulation. Students randomised into the control arm received the seminars and workshops alone. At the end of each day of the simulation, students in both arms were asked to submit a short reflective journal detailing their learning experiences and at the end of the week they complete a range of trial-specific assessment items. Comparison of the two groups showed that CLEIMS had significant and persistent positive impact on students’ prescribing skills, among other benefits in the cognitive and psychomotor domains ( Rogers, McConnell, Jones de Rooy, Ellem, & Lombard, 2014). The authors also noted that other benefits of CLEIMS appeared to permeate students’ reflective journals, such as improved confidence and development of professional values, consistent with learning in the affective domain. However, verifying and quantifying this learning posed significant challenge due to the lack of a validated, reliable and practical instrument, particularly within health professional education.

Recognising the importance of health practitioners acquiring affective learnings from emotionally-impactive experiences, we further examined the students’ reflective journals to determine whether CLEIMS had indeed enhanced their affective learning when compared with the non-simulation-based control experience alone. During this process, it became clear that to be able to identify evidence of affective learning, we first needed to be able to interpret students’ reflective accounts of their learning experiences. We explored the use of interpretative phenomenological analysis (IPA), a method originally developed by Jonathan Smith (1996) to identify the meanings derived from the experiences of people living with chronic or serious health conditions. Smith, in collaboration with Mike Osborn, proposed that a ‘double hermeneutic’ process be applied when interpreting texts or transcripts ( Smith & Osborn, 2008). That is, the analyst first examines the way in which the learner has made sense of the experience that they described (the first hermeneutic), followed by a reflective process in which the analyst attempts to make sense of the respondent’s sense-making in psychological terms (the second hermeneutic). The use of this method enabled the affective learning for which there was evidence in each journal entry to be categorised according to Krathwohl’s hierarchy (1964).

The development of our phenomenologically-derived method to assess affective learning in student journals was recently reported in *Medical Teacher* ( Rogers, Mey, & Chan, 2017). We demonstrated that the technique, based on the IPA methodology, appeared to be robust for assessing for the presence and quality of affective learning among medical students. We also showed that participation in the full CLEIMS immersive simulation was associated with higher level affective learning, as measured by this method, when compared with related conventional seminars and workshops alone. We recognised that the development of an ordinal ranking of learning achievement from the application of a phenomenological method ‘might be considered highly transgressive’ but argued that it was an example of ‘ability to work nimbly in the borderlands between [positivist and human science] paradigms’ that is ‘a critical capability for effective health professional educators’ ( Rogers, Mey, & Chan, 2017, p. 1259).

The assessment of professionalism has been described as a ‘wicked problem’ (Varpio, Aschenbrener, & Bates, 2017) and a recent international consensus statement described the acquisition of interprofessional values, in particular, as ‘difficult to measure directly’ ( Rogers, Thistlethwaite, et al., 2017, p 352). In order to fill this gap in the assessment armamentarium, we sought to translate our IPA-derived technique into a practical tool that could be used along with reflective journaling to assess affective learning reliably. We postulated that this would enable educators to verify that affective learning associated with the acquisition of professional values was taking place and, if used summatively, would convey to learners the importance attached to this area of capability by the professions and by the community.

We noted in the prior study ( Rogers, Mey, & Chan, 2017) that, at the higher levels of affective learning, it was challenging to categorise some reflections where there was evidence of learning beyond the organisation level, but insufficient to be described at the characterisation level since there was not yet confirmation of its ongoing impact on practice. Indeed, the study team recognised a transition phase where students had appeared to incorporate the affective learning brought about by the experiences into their value system and had committed to integrating this learning into their ongoing practice but had not yet had the opportunity actually to do so. Thus, when we derived a numerical scale based on Krathwohl’s levels of affective learning, in the current study, we added a transitional level between his original descriptions of ‘organisation’ and ‘characterisation’.

## Method

Based on the findings of our earlier study ( Rogers, Mey & Chan, 2017), we developed a single seven-point Likert-type instrument that we dubbed the Griffith University Affective Learning Scale (GUALS). To Krathwohl’s original five levels (receiving [designated as ‘2’], responding [‘3’], valuing [‘4’], organisation [‘5’] and characterisation [‘7’]) we added two further points: ‘no affective learning’ (designated ‘1’ on the Likert-type scale) and a further point (designated ‘6’) between organisation and characterisation. The ‘6’ level on the scale was intended to apply where the affective learning associated with the experience appeared to have been incorporated into the learner’s value system, this incorporation appeared authentic on the application of the double hermeneutic method and there was evidence of a commitment to the its application in future practice, but this commitment was yet to be fulfilled. Full details of the method and examples from journals where affective learning was evaluated to be at each of Krathwohl’s levels are provided in our earlier publication ( Rogers, Mey & Chan, 2017).

**Figure 1.**
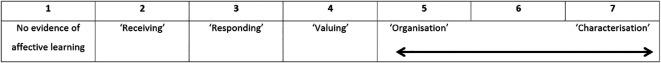
The GUALS Likert-type Scale.

In the current study, we designed a paper-based tool that incorporated the scale, along with *aides mémoire* descriptors for each level and an exhortation to assessors to rate the *highest* level of affective learning for which they could find authentic evidence on the application of the double hermeneutic method. The GUALS Likert-type scale is depicted in Figure 1. We have not reproduced the full instrument here, as it is utilised only by assessors and not disclosed to learners. This is because of theoretical concerns about the likelihood of students (especially highly capable health professional students) ‘writing to the rubric’ ( Mabry, 1999) and thus tending to invalidate the scale as a means to assess authentic affective learning. Readers who are interested in using the instrument are invited to contact the first author directly to obtain a copy of the full GUALS instrument and details of the conditions of its use.

We trained seven learning facilitators in the assessment of affective learning by the method outlined in our prior publication ( Rogers, Mey & Chan, 2017) and the utilisation of the tool. The facilitators were senior intensive care or emergency department nurses who were experienced in facilitation of learning in the CLEIMS methodology. The training took approximately one hour and comprised discussion of the concept of affective learning and Krathwohl’s levels, as well as the double hermeneutic technique. This was followed by introduction to the scale and practice in blinded rating of multiple exemplar journals, then detailed discussion of the ratings made and the reasoning that led to them.

**Figure 2.**
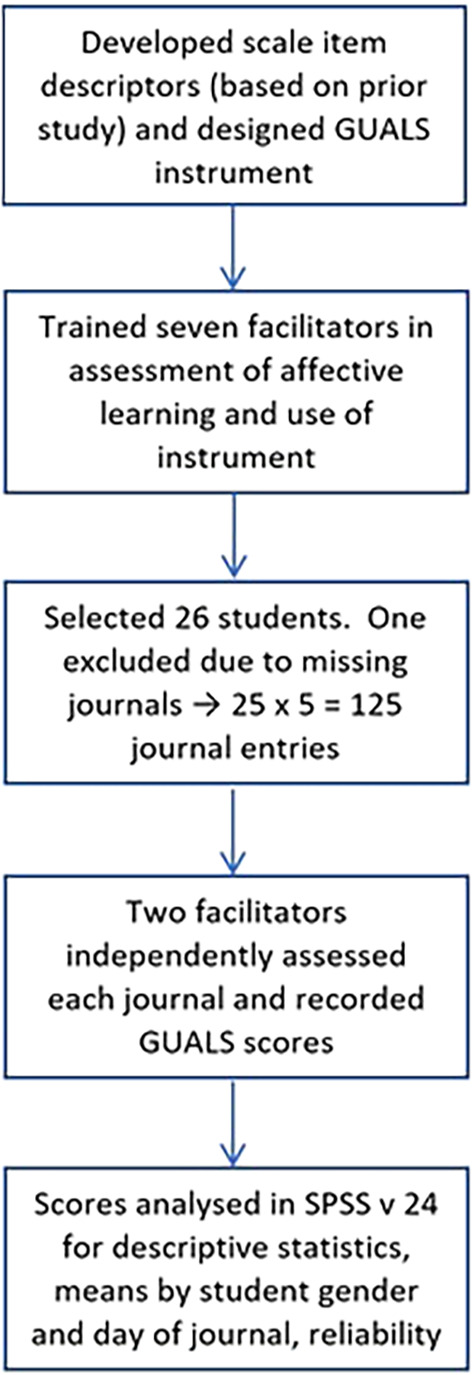
The study design.

A convenience sample of 26 students from the most recent offering of CLEIMS was then selected and their journals were assessed by the trained facilitators. Each entry was assessed independently by two assessors. The resulting scores were analysed utilising IBM® SPSS® Statistics, version 24, to yield descriptive statistics, a comparison of mean scores in relation to student gender and the day of the CLEIMS week to which the journal referred, as well as reliability analysis of the concordance between facilitator scores. The study design is summarised in Figure 2. Under the guidelines of our local human research ethics committee, this work was considered to be evaluation of an assessment technique and thus did not require explicit ethics committee approval or student consent. The assessing facilitators volunteered to take part in the study.

## Results

A total of 26 (female = 13, male = 13) penultimate year medical students had recently completed the CLEIMS week at the time of this study, each submitting five daily reflective journals, which would have provided a total of 130 journals for rating. However, one participant failed to submit one journal and so that student’s data were excluded from analysis. Thus, 125 journal entries were each rated independently by two of the seven trained facilitators.

### Students’ performance

The lowest level recorded was a ‘1’ on the seven-point GUALS scale and the highest score achieved was a ‘6’. The mean scores achieved across the days of the CLEIMS week are presented in Table 1 according to student gender.

**Table 1.**
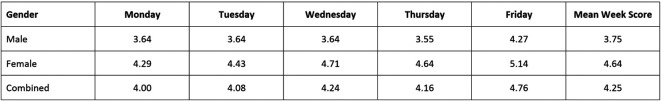
Students’ mean GUALS scores across the CLEIMS week

The highest mean score was achieved after the final day of CLEIMS for both male and female participants. T-tests to compare the daily mean scores revealed that female students demonstrated significantly higher levels of affective learning than their male counterparts in journals from Wednesday ( *p* = 0.018) and Thursday ( *p* = 0.021), as well as overall across the CLEIMS week ( *p* = 0.023). Mean scores for the Friday journals were significantly higher than those for Monday journals in both genders (male *p* = 0.026, female *p* = 0.012).

### Reliability

**Figure 3.**
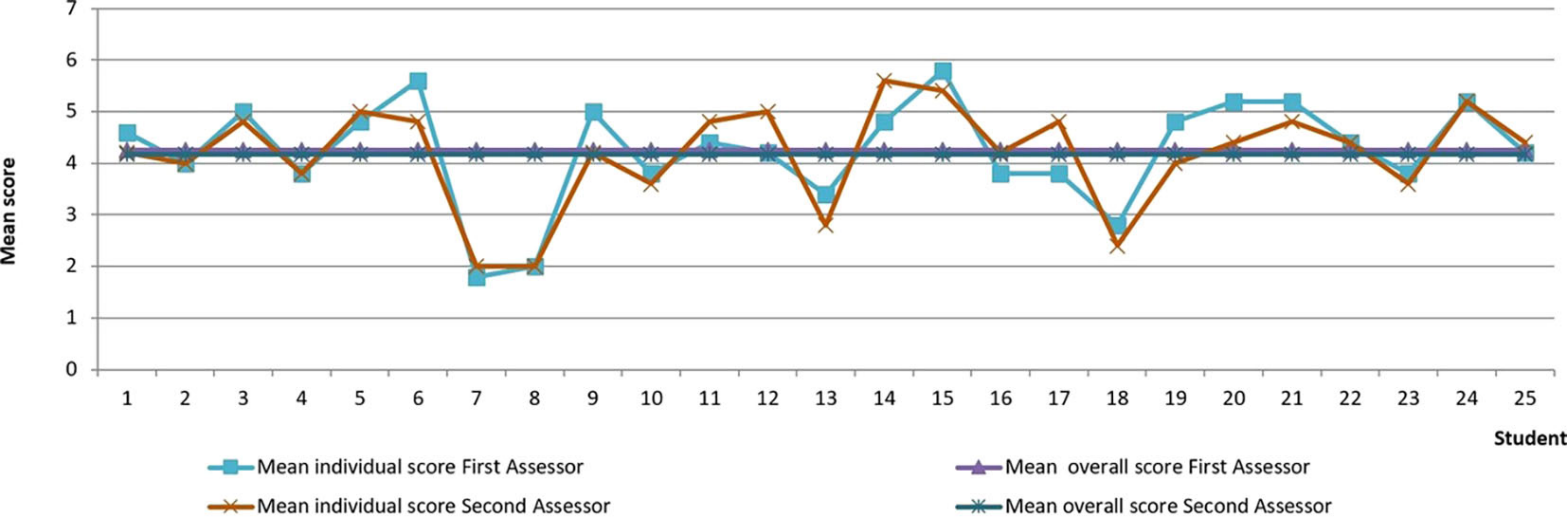
Distribution of GUALS scores.

The distribution of mean scores awarded by the two assessors for each student’s journals is presented in Figure 3.

The mean score awarded by the first assessor of each journal was 4.25 ± 1.20 on the seven-point scale and the mean score awarded by the second assessor was 4.17 ± 1.16 (displayed as horizontal lines in Figure 3). There were no significant differences between mean scores awarded by the two assessors (t = 1.079, df =124, *p* = 0.283). Inter-rater absolute concordance was found in 57 of the 125 journals (45.6%). The mean inter-rater difference was 0.59 points and the maximum difference was two points, which occurred in less than 5% of all cases. The intraclass correlation coefficient (ICC) was 0.86 (95% Cl: 0.80 - 0.90).

## Discussion

While recognition of the importance of affective learning is growing, especially in relation to the acquisition of professional and interprofessional values ( Brown, Ferrill, Hinton, & Shek, 2001; Buissink-Smith, Mann, & Shephard, 2011; Einhellig, Hummel, & Gryskiewicz, 2015; Rogers, Thistlethwaite, et al., 2017), assessment within this domain has been challenging, due mainly to a dearth of validated and reliable tools. We recently published a method, informed by IPA and mapped to Krathwohl’s levels, for the assessment of affective learning in the journals of learners who have participated in emotionally-impactive learning experiences ( Rogers, Mey & Chan, 2017). In that publication we acknowledged that further work was needed to develop a practical scale that educators could be trained to apply reliably. In the current paper, we have reported on the development of such a tool and an initial study to investigate its reliability for medium-stakes summative assessment of health professionals.

We found that the use of the GUALS on medical student reflective journals by trained assessors demonstrated a level of inter-rater reliability (ICC = 0.86 [95%CI: 0.80 - 0.90]) that Downing (2004) has described as acceptable for medium to high stakes assessments of health professionals. The detailed analysis in our prior study speaks to the face validity of the general approach as a measure of affective learning. The present finding that learners’ GUALS scores improved across the week also suggests that the instrument can detect a form of learning that improves with increased experience of reflection on practice challenges. The finding that the journals of female medical students scored higher across the study than those of their male counterparts also may also speak to validity in that it accords with the traditional view that women generally engage more easily and more effectively with the emotional and human dimensions of learning experiences than men.

Given that a significant proportion of the affective learning we observed in our prior study related to interprofessional values and a worldview that supports collaborative practice, the development of this instrument may partly answer the call articulated in the recent International Consensus Statement on the Assessment of Interprofessional Learning Outcomes for ‘further work’ on the ‘assessment of reflective journaling to identify affective learning’ in this area ( Rogers, Thistlethwaite et al., 2017, p 356).

This study’s findings are limited by the fact that it evaluated the journals of a relatively small number of learners from a single health profession. It also sought to measure affective learning that had arisen in association with only a single (albeit complex and extended) learning activity, in a simulated rather than a real practice setting. Further, the inter-rater reliability of the tool was measured soon after the raters had received a one-hour practical training session in its use. It is yet to be determined whether the good reliability obtained would be maintained over time in the absence of further periodic training or calibration. We detected a significant difference between the scores of women and men, which may reflect a true difference in affective learning but could, in part, be an artefact associated with other gender differences such as general writing capability ( Fearrington, et al. 2014).

Future work should investigate the utilisation of the GUALS in relation to learning experiences in other settings such as real clinical practice. It could also investigate its applicability for measuring the affective learning of other health professionals and even a wider range of occupations where affective learning is important such as police officers and military personnel. The instrument might also be trialled in relation to the affective development of educators such as school teachers, as well as the small-group facilitators and simulated patients who themselves support the learning of health professionals. We have assumed on theoretical grounds that providing the instrument in advance to the learners whose journals were to be assessed might adversely affect the validity of the assessment approach if they ‘write to the rubric’ ( Mabry, 1999). For this reason, we have not published the complete instrument here but invite readers to contact us directly if they are interested in trialling the technique. It would also be interesting to conduct a prospective study to determine whether this concern is justified, as well as to investigate the impact of providing progressive feedback to learners on the affective content of their journals. Finally, since the tool and associated assessment technique rely on the detailed interpretation of students’ writing in the English language, it would be of value to study their applicability for learners within other cultural traditions or those who are writing in a second language.

## Take Home Messages

• Learning in the affective domain of Bloom’s Taxonomy is closely related to the development of professionalism and interprofessional values in health professional education but is difficult to measure
• The application of a method based on interpretative phenomenological analysis enables assessment of the presence and quality of affective learning in the reflective journals of health professional students who have engaged in emotionally-impactive learning experiences
• The Griffith University Affective Learning Scale (GUALS) appears to be a reliable tool for the measurement of affective learning by this method when applied by trained assessors.

## Notes On Contributors

***Gary D. Rogers***, MBBS, MPGPsych, PhD, FANZAHPE, FAMEE, PFHEA is Professor of Medical Education and Deputy Head (Learning & Teaching) of the School of Medicine at Griffith University, in addition to a role as Program Lead for Interprofessional and Simulation-Based Learning in the Griffith Health Institute for the Development of Education and Scholarship (Health IDEAS).

***Amary Mey***, BPharm(Hons), PhD, is a Research Fellow at the School of Pharmacy and Pharmacology, and Health IDEAS, Griffith University, Gold Cost, Australia.

***Pit Cheng Chan***, BPharm, MSc, GCertHigherEd, is Coordinator of Interprofessional and Simulation-Based Learning in Health IDEAS at Griffith University.

***Marise Lombard***, MSocSci, RN, RM, IPN, NMPSP, is Lecturer in Medical Education and a PhD candidate in the School of Medicine, Griffith University.

***Fiona Miller***, BPharm, is Lecturer in Pharmacy Education in the School of Pharmacy and Pharmacology, and Health IDEAS, Griffith University.
