# Supplementary material for: Development and validation of the Griffith University Affective Learning Scale (GUALS): A tool for assessing affective learning in health professional students’ reflective journals
Source: MedEdPublish (2016). 2018 Jan 4;7:2. [Version 1] doi: 10.15694/mep.2018.000002.1 (PMC10701821; doi:10.15694/mep.2018.000002.1)
Supplement: Peer review history

# Reviewer report: Reviewer response for version 1

**DOI:** 10.21956/mep.19475.r28744

**Published:** 2018-07-18

**Authors:** Michaela Kelly

**Affiliations:** 1 University of Queensland

This review has been migrated. The reviewer awarded 3 stars out of 5 Thank you for this very interesting and well written article. The GUALS is a worthwhile instrument and provides a clear means to appraise affective learning in medical students. My only reservation is that the rubric is not disclosed to students. I understand the argument to prevent students from ‘gaming’ their reflections however, I wonder whether students having visibility of the rubric is a learning experience in itself. I personally believe that if work is assessed it is important that students understand how that work will be appraised.

Reviewer Expertise:

NA

No decision status is available

# Reviewer report: Reviewer response for version 1

**DOI:** 10.21956/mep.19475.r28742

**Published:** 2018-01-09

**Authors:** Mohammed Osman

**Affiliations:** 1 General orgainization for teaching hospitals

This review has been migrated. The reviewer awarded 3 stars out of 5 Overall, it is an interesting manuscript. It has a clear objective which was to develop and evaluate the reliability of an instrument assess for evidence of affective learning in the reflective journals for students who participated in emotionally-impassive learning experiences through developing a single seven-point Likert-type GUALS scale.The language was easy for readers. The number of ratings was fine. The reliability was assessed by 2 independent assessors to minimise bias.However, the manuscript lacks a concise conclusion. Also, the number of participants was small to generalize these results especially it seems they were from the same site and the same year. Therefore, I believe more confirmatory trials are recommended. As well as that, reliability is only one point to evaluate new tools. They include their educational impact, credibility, feasibility and their cost.

Reviewer Expertise:

NA

No decision status is available

# Reviewer report: Reviewer response for version 1

**DOI:** 10.21956/mep.19475.r28743

**Published:** 2018-01-09

**Authors:** Johnny Lyon Maris

**Affiliations:** 1 Southampton GP Education Unit

This review has been migrated. The reviewer awarded 4 stars out of 5 This is an interesting and growing area of student development. Capturing assessment of professionalism is important and therefore this paper adds support and weight to the literature in this area. It is well written with clear descriptions of what the authors were looking for and the science behind it. In addition to the results, the challenges with the work are recognised clearly. The authors , as stated, have not published the tool but it is available on personal contact with the authors. I have no reservations in recommending this article.

Reviewer Expertise:

NA

No decision status is available

# Reviewer report: Reviewer response for version 1

**DOI:** 10.21956/mep.19475.r28745

**Published:** 2018-01-04

**Authors:** Trevor Gibbs

**Affiliations:** 1 AMEE

This review has been migrated. The reviewer awarded 5 stars out of 5 A very interesting paper to read; well researched and well written and one that covers very important area of student learning.The issue of assessment in the affect domain, as clearly described by the authors, remains a major issue and this piece of research adds very positively to our toolbox of appropriate measuring tools. I particularly liked the logical approach the authors used in their research and in writing the paper- I feel that many new researchers could benefit from learning how this has been structured through systematic enquiry, leading to logical steps in research

Reviewer Expertise:

NA

No decision status is available
